# Rapid screening of staphylokinase protein variants using an unpurified cell‐free expression system

**DOI:** 10.1002/2211-5463.70229

**Published:** 2026-03-24

**Authors:** Maria Tomková, Veronika Hovanová, Jiri Damborsky, Erik Sedlák

**Affiliations:** ^1^ Center for Interdisciplinary Biosciences P. J. Šafárik University in Košice Slovakia; ^2^ Department of Biochemistry, Faculty of Science P. J. Šafárik University in Košice Slovakia; ^3^ Loschmidt Laboratories, Department of Experimental Biology and RECETOX Masaryk University Brno Czech Republic; ^4^ International Clinical Research Centre St. Anne's University Hospital Brno Czech Republic

**Keywords:** cell‐free protein synthesis, chromogenic assay, directed evolution, protein screening, staphylokinase, thrombolytics

## Abstract

Protein engineering approaches, including rational design and directed evolution, are essential for optimizing protein properties in biotechnology. However, their application is often limited by the need to experimentally characterize large numbers of variants. This is especially true for directed evolution, where selected candidates require heterologous expression and purification before functional testing. To reduce this bottleneck for staphylokinase, a fibrin‐specific thrombolytic with therapeutic potential, we developed a rapid screening platform based on cell‐free protein synthesis (CFPS) and a chromogenic plasminogen‐activation assay. Activity rankings from crude CFPS mixtures closely matched those from purified proteins, showing that the method provides reliable functional readouts without purification. This CFPS‐based workflow offers a fast, scalable, and efficient solution for early‐stage screening of staphylokinase variants and can accelerate the identification of new thrombolytic candidates. The modularity of this method also allows its facile adjustment for other enzymes.

## Graphical abstract

An unpurified cell‐free protein synthesis (CFPS) platform enables rapid functional screening of staphylokinase variants. Direct plasminogen‐activation assays performed in microplate format provide real‐time activity readouts, allowing rapid identification and ranking of variants with improved or reduced fibrinolytic activity without protein purification. Created in BioRender. Tomkova, M. (2026) https://BioRender.com/gkupf0w.

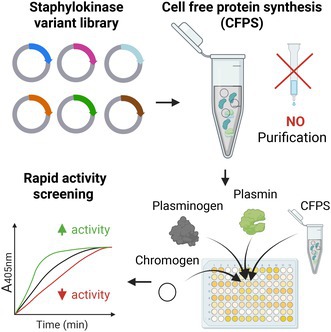

**Abbreviations**
CFPS: cell‐free protein synthesis
PLG: plasminogen
PLM: plasmin
PUREfrex: reconstituted cell‐free transcription–translation system
SAK: staphylokinase

## Introduction

Natural proteins represent an important class of molecules with significant potential for industrial applications [1]. Although proteins function efficiently in their native environments, industrial processes or other applications in pharmacology or biotechnology often require modifications to enhance their performance, including improved stability, activity, or specificity [2, 3, 4]. Protein engineering strategies include rational design and directed evolution methods. Directed evolution is a powerful approach that mimics Darwinian evolution in the laboratory by iterative cycles of mutation and selection, enabling the improvement of protein properties [5]. This approach relies on selection‐ and screening‐based methods that facilitate effective identification and enhancement of variants with desirable traits. Despite its success, a major bottleneck in directed evolution and rational design lies in the experimental characterization of selected variants, especially for assessing enzyme stability and catalytic activity [6]. Functional assays typically require heterologous protein production, and often purification, which are labor‐intensive steps that significantly limit the throughput and scale of screening.

We focus on staphylokinase (SAK) research, an extracellular bacterial protein with fibrinolytic activity that has emerged as a promising thrombolytic agent, functioning as a plasminogen activator [7]. SAK itself is enzymatically inactive, and it works as a “cofactor” by forming a complex with plasmin [8]. This SAK–plasmin complex subsequently binds and autocatalytically activates plasminogen. Unlike many other thrombolytics, SAK‐mediated plasminogen activation is fibrin‐specific, which helps minimize systemic bleeding risks [7]. Despite its therapeutic potential, several limitations hinder clinical application of SAK, including high immunogenicity, rapid clearance from circulation, and modest catalytic efficiency [9]. Among the factors responsible for these drawbacks is SAK's moderate affinity for plasmin, which limits the formation of an active plasmin–SAK complex and consequently diminishes its effectiveness [10].

**Fig. 1:**
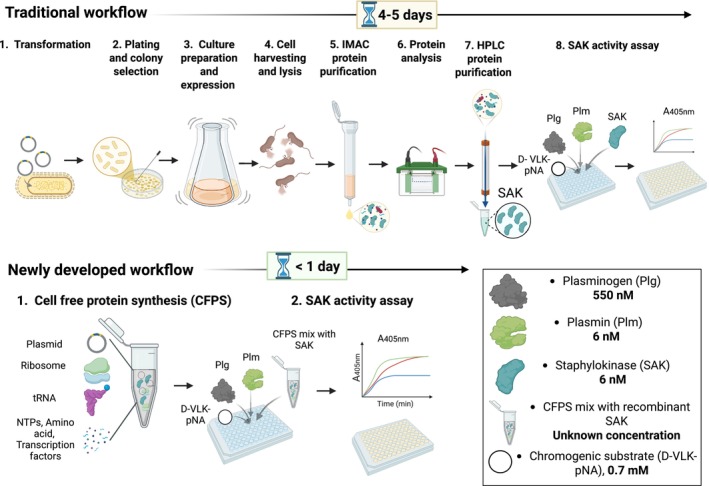
Schematic overview of traditional and CFPS‐based workflows for SAK variant activity screening. The traditional workflow is based on conventional protein production, isolation, and purification, whereas the newly developed PUREfrex workflow combines CFPS with a direct chromogenic activity assay. The traditional approach, aiming to test the activity with the purified protein, consists of multistep processes and involves significantly more effort and material and usually takes 4–5 days. In comparison, the PUREfrex is a process consisting of only a few steps, involving a mixture of the reagents, performing measurement of the chromogenic assay and the result analysis, which can be accomplished within 5–6 h. Plasminogen (Plg), plasmin (Plm), and the chromogenic substrate S‐2251 are used to monitor plasmin generation by the SAK–plasmin complex via absorbance at 405 nm. Created in BioRender. Tomkova, M. (2026) https://BioRender.com/7brtb5l.

To address this, we used ribosome display, a directed evolution method, to improve the plasmin‐binding properties of the naturally occurring SAK variant 42D through affinity maturation. This approach produces many SAK variants that need functional testing. Because plasminogen activation by SAK is a multistep process, better plasmin binding does not always mean higher activity, so a proper functional assay is required. Chromogenic activity assays are well‐established methods for measuring the activity of plasminogen activators [11, 12, 13, 14, 15]. They provide sensitive, quantitative, and time‐efficient detection of plasmin generation. Currently, chromogenic assays, as well as other thrombolytic profiling methods, rely on recombinant protein production [15]. To simplify this process, we developed a screening method using cell‐free protein synthesis (CFPS). CFPS rapidly produces proteins *in vitro* using the cellular translational machinery and offers broad versatility for applications in biotechnology, synthetic biology, enzyme engineering, and diagnostics [16]. By combining CFPS with a chromogenic assay, we achieved direct functional assessment of SAK variants without the need for bacterial expression or purification (Fig. 1). This type of CFPS‐based functional thrombolytics testing has not been used before, providing a faster route for early activity‐based screening.

## Material and methods

### Plasmid information

Genes encoding SAK variants were cloned into pQE‐30 (T5 promoter, ampicillin resistance) for expression of N‐terminal 6 × His‐tagged proteins or into pET‐28b (+) (T7 promoter, kanamycin resistance) for expression in the PUREfrex cell‐free system.

### SAK variants

All variants used in this study were derived from the wild type SAK 42D and were selected using a directed evolution approach based on ribosome display. The individual protein codes represent internal designations that reflect the selection round and the sequenced variant.

### Transformation and culture

SAK proteins were expressed and purified as previously described, with minor modifications [17]. SAK genes were cloned into the pQE‐30 vector using the BamHI and HindIII restriction sites. Plasmids (50 ng of DNA) were transformed into 50 μL of chemically competent *E. coli* BL21 (DE3) cells using the heat shock method (45 s at 42 °C). Cells were recovered in 500 μL of 2 × YT medium for 30 min at 37 °C with shaking at 900 rpm and then plated on 2 × YT agar plates supplemented with 100 μg·m^−1^ ampicillin. Transformants were used to inoculate overnight cultures grown for 18 h at 37 °C, 250 rpm, in 10 mL of 2 × YT medium containing 100 μg·m^−1^ ampicillin. These cultures served as the inoculum for expression cultures, which were grown in 2 × YT medium supplemented with 100 μg·m^−1^ ampicillin and diluted to an OD_600_ of 0.1. Expression cultures were grown at 37 °C with shaking at 180 rpm until the OD_600_ reached 0.6–0.8, at which point protein synthesis was induced with 0.1 mM isopropyl β‐d‐1‐thiogalactopyranoside (IPTG). Proteins were expressed for 18 h at 20 °C with shaking at 180 rpm. Bacterial cultures were centrifuged, and cell pellets were flash‐frozen in liquid nitrogen and stored at −80 °C.

### SAK purification

Cell pellets were resuspended in a lysis buffer containing 50 mm HEPES, 200 mm NaCl, 1 mm EDTA, 7 mm lysozyme, and protease inhibitors (10 μm leupeptin and 1 μm pepstatin), prepared freshly and kept on ice. Cells were lysed by sonication using a Branson Sonifier 450 (Thermo Fisher Scientific, USA). The lysates were centrifuged at 17500 × ***g*** for 30 min at 4 °C, and the supernatants were applied to a gravity column containing Ni‐NTA resin (Thermo Fisher Scientific, Waltham, MA, USA) equilibrated with 50 mm HEPES and 200 mm NaCl. After loading, the column was washed with buffer containing 50 mm HEPES, 200 mm NaCl, and 40 mm imidazole to remove non‐specifically bound proteins. His‐tagged proteins were eluted with buffer comprising 50 mm HEPES, 200 mm NaCl, 250 mm imidazole, and 10% glycerol. Eluted proteins were concentrated using Amicon Ultra centrifugal filters with a 10 kDa molecular weight cutoff (Merck, Germany) and further purified by size‐exclusion chromatography on a Superdex 75 Increase 10/300 GL column equilibrated with 50 mm phosphate buffer (pH 7.5). Proteins were eluted under isocratic conditions at a flow rate of 0.5 mL·min^−1^. Elution was monitored by UV absorbance at 280 nm, and the chromatographic run lasted approximately 60 min (Fig. S1). Fractions corresponding to the major protein peak were collected and analyzed by 15% SDS/PAGE, followed by staining with InstantBlue Coomassie stain (Sigma‐Aldrich, St. Louis, MO, USA). Fractions with the highest purity were pooled, flash‐frozen in liquid nitrogen, and stored at −80 °C.

### Protein synthesis using the PUREfrex cell‐free system

Cell‐free protein synthesis was carried out using the PUREfrex cell‐free transcription‐translation system (GeneFrontier, Kashi, Japan) following the manufacturer's protocol with minor modifications. SAK genes were cloned into the pET‐28b(+) plasmid using the NcoI and XhoI restriction sites. This vector contains a T7 promoter, a ribosome‐binding site, and the start and stop codons necessary for acellular protein production. The total amount of DNA template in each reaction was 350 ng. Each 20 μL reaction mixture consisted of 10 μL of Solution I, 1 μL of Solution II, 2 μL of Solution III, the DNA template, and nuclease‐free water to reach the final volume. All components were gently mixed by pipetting, while avoiding vortexing. All steps were performed to maintain an RNase‐free environment. Reactions were incubated at 37 °C for 3.5 h without shaking. Control reactions without a DNA template were included to assess background expression. For the subsequent activity assays, which were performed in triplicates, the reaction mixture was diluted with 12 μL of SAK activity assay reaction buffer to ensure sufficient volume and optimal measurement conditions. Immediately following incubation, 10 μL of this diluted mixture was used for the SAK activity assay.

### SAK activity assay

The activity of SAK variants was determined using a chromogenic assay for plasminogen activation [15, 18, 19]. *Reaction buffer*. The reaction buffer consisted of phosphate‐buffered saline (PBS) supplemented with 1 mm CaCl₂ and 0.1% (v/v) Tween 80. The final pH was adjusted to 7.4, and the buffer was stored at 4–8 °C. *Proteins and reagents*. Lyophilized human plasmin (81 kDa; Athens Research & Technology, Athens, GA, USA) was reconstituted in sterile water to a final concentration of 1 mg·mL^−1^. Lyophilized human plasminogen (90–94 kDa; Roche Diagnostics) was reconstituted in sterile water to a final concentration of 1 mg·mL^−1^. All protein stock solutions were aliquoted to prevent repeated freeze–thaw cycles and stored at −80 °C. Thawed aliquots were used once and not refrozen. All working dilutions were prepared freshly in the reaction buffer before each experiment. *Activity assay*. The reaction mixture was prepared to a final volume of 100 μL by combining 10 μL of SAK (final concentration 6 nm), 20 μL of plasmin (final concentration 6 nm), 50 μL of plasminogen (final concentration 550 nm), and 20 μL of S‐2251 chromogenic substrate (0.7 mm; D‐VLK‐pNA·2HCl; DiaPharma, West Chester, PA, USA). All components were diluted in the reaction buffer prior to their addition to the reaction mixture. Assays were performed at 37 °C using transparent flat‐bottom 96‐well microplates (Nunclon™ Delta Surface; Thermo Fisher Scientific, USA). Before the initiation of the reaction, plasmin and SAK were pre‐incubated for 30 min at 37 °C to facilitate complex formation. Following pre‐incubation, the chromogenic substrate was added, and the reaction was initiated by the addition of plasminogen. For the cell‐free synthesis assay, 10 μL of the PUREfrex reaction mixture (GeneFrontier Corporation, Kashiwa, Japan) was added to the assay in place of the purified SAK protein. The precise concentration of SAK within the PUREfrex mixture was not determined due to its low abundance and the presence of endogenous system proteins. Kinetic measurements of absorbance at 405 nm were performed at 20–30 s intervals for 40 min using a Synergy HTX multi‐mode microplate reader (BioTek Instruments, Agilent Technologies, Santa Clara, CA, USA). Negative controls were included in every experimental run. For assays involving purified proteins, the negative control contained the complete reaction mixture, omitting the protein components. For the PUREfrex‐based assays, the negative control consisted of a PUREfrex reaction mixture prepared without the addition of a DNA template.

### Data analysis

Enzymatic activity was quantified by calculating the reaction half‐time (τ_1/2_), which was defined as the time required to reach 50% of the maximum absorbance.

## Results and discussion

**Fig. 2:**
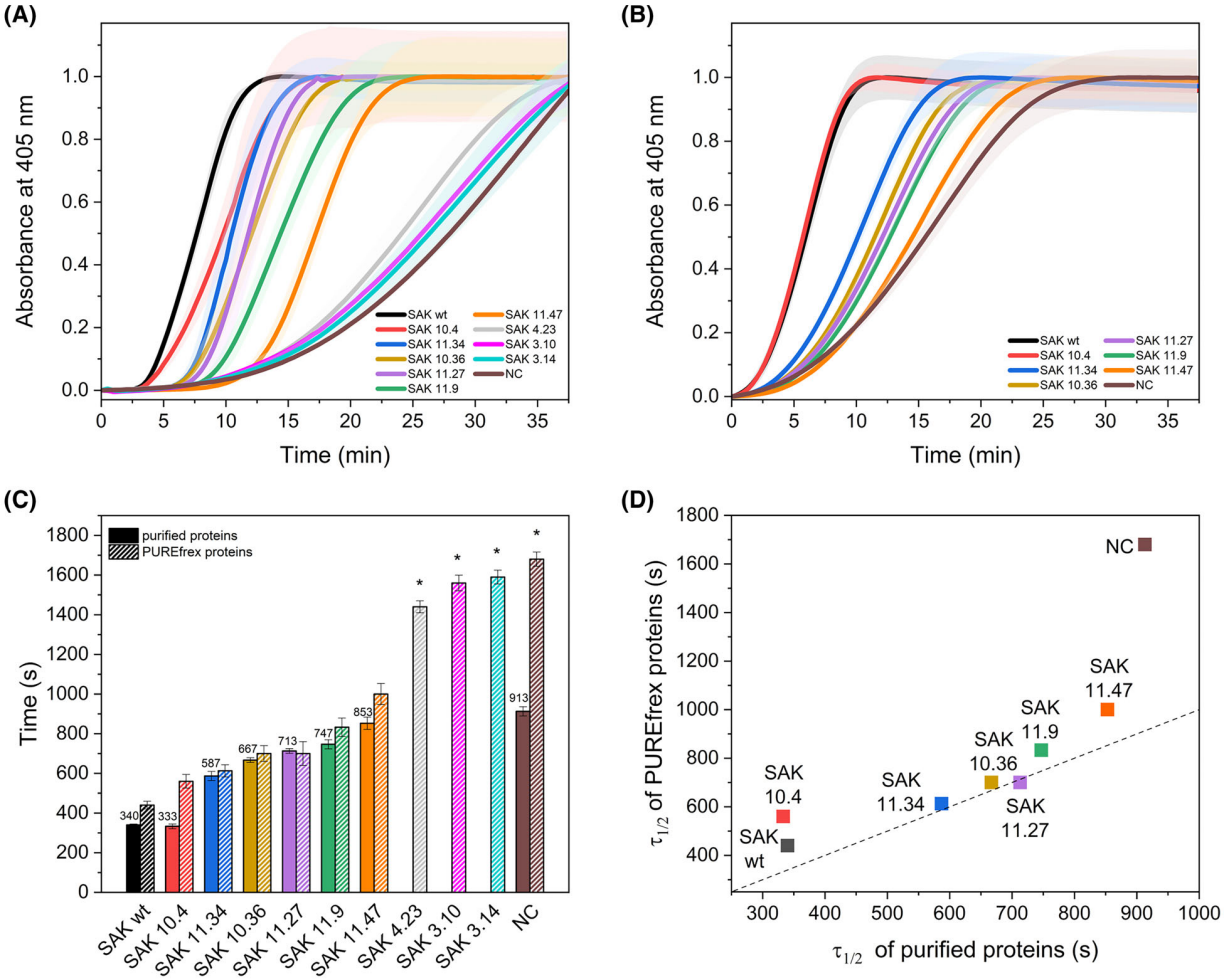
Results of the SAK activity chromogenic assay using PUREfrex crude reaction mixtures (A) and purified proteins (B). Reaction curves represent the average of three replicates for each protein. The filled area corresponds to the standard deviation of the kinetic data. (C) Variant ranking based on the τ₁/₂ evaluation method. Data marked with an asterisk (*) represent a lower estimate of τ₁/₂, as the kinetics did not reach a plateau. Error bars in panel C represent standard deviations of triplicates. (D) Correlation between the half‐times (τ₁/₂) obtained for purified proteins and those measured in PUREfrex crude reaction mixtures. Each data point represents one SAK variant (or NC), and the dashed line indicates the line of identity. Error bars represent the standard deviations of triplicates for pure proteins (−) and proteins from PUREfrex reaction mixture (I). The individual protein codes represent internal designations corresponding to the ribosome display selection round and the sequenced variant.

We evaluated a newly developed chromogenic assay on nine SAK variants with distinct activities by testing both the crude PUREfrex cell‐free mixtures (Fig. 2A) and the purified proteins (Fig. 2B).

To compare the results, we used the reaction half‐time (τ_1/2_), a simple measure of enzymatic activity indicating how quickly the reaction reaches its midpoint ‐ shorter τ_1/2_ values reflect faster activity (Fig. 2C). This analysis produced consistent data for both purified proteins and crude PUREfrex expression mixtures in both followed parameters: (a) the relative ranking of the SAK‐induced plasmin activities and even (b) comparable reaction half‐times of corresponding SAK. In fact, the plot shown in Fig. 2D clearly indicates a strong correlation between τ_1/2_ values obtained by activity measurements using purified proteins by traditional methods and activity obtained by proteins expressed by the PUREfrex method.

In the assay with PUREfrex reaction mixture (Fig. 2A), the order of activity was as follows: wild type > 10.4 > 11.34 > 10.36 ≈ 11.27 > 11.9 > 11.47 > 4.23 > 3.10 > 3.14. The protein‐based assay (Fig. 2B) yielded a broadly similar ranking, with only a minor shift in the performance of variant 10.4: 10.4 ≈ wild type > 11.34 > 10.36 > 11.27 > 11.9 > 11.47. The activities of variants 4.23, 3.10, and 3.14 were detected only in the PUREfrex assay, where their activities were slightly higher than the negative control. Purified proteins of these variants could not be obtained using conventional expression systems due to the very low protein production level. We emphasize that the variants analyzed here were selected primarily to illustrate the applicability of our methods. In practice, directed protein evolution typically yields many variants to be characterized and used for further rounds of evolution.

In the case of PUREfrex protein synthesis system, the expressed protein contained only three additional N‐terminal amino acids (MGM) preceding the native SAK sequence. In contrast, the protein purified from *E. coli* carried a longer N‐terminal extension (MRGSHHHHHHGSM) due to the His_6_ affinity tag required for purification. As demonstrated in previous studies [20, 21, 22], the presence of a His‐tag may affect the biophysical properties of proteins. We showed very recently that in the case of SAK variants, the His‐tag slightly reduces protein activity [17]. This difference could have led to minor variations in the relative protein ranking between the two assays. Short three amino acids N‐terminal tag in the PUREfrex proteins, moreover in the mixture containing all additives necessary for protein translation, apparently has no significant impact on the overall comparative analysis in the case of SAK variants, as clearly follows from the strong correlation shown in Fig. 2D.

The PUREfrex‐based assay provides a composite readout that integrates SAK catalytic activity with CFPS expression efficiency, thereby highlighting its advantage for rapid functional screening of variants. Using the PUREfrex system, variants that fail to express or express only weakly can be quickly assessed at an early stage, while those combining strong expression with biological activity are prioritized for further experimental studies. Although this dual readout may miss variants that are highly active but poorly expressed in PUREfrex, such a compromise aligns well with the practical requirements of large‐scale protein production, where both activity and expressibility are essential [23]. From a biotechnological perspective, this feature represents an advantage compared to traditional workflows that require time‐consuming purification and characterization of every candidate.

Although this short note focused solely on SAK, the workflow combining PUREfrex expression with chromogenic assays can be adapted for other targets with particular advantage in enzyme selection. Two recent studies have shown that CFPS can be adapted for various screening purposes. For example, it has been combined with a colorimetric assay to screen azoreductase variants and identify their preferred electron donor [24], and with binding assays to rapidly express and screen antibody fragments against SARS‐CoV‐2 [16]. Both studies, like ours, show that CFPS can bypass laborious purification and enable direct functional readouts. CFPS represents a robust method in identifying promising candidates in directed protein evolution for further development by methods of protein engineering.

## Conclusions

In summary, our study demonstrates that CFPS provides an effective, simple, and fast platform for the rapid screening of SAK protein variant activities. While the method is well‐suited for comparing relative activities across multiple samples, classical protein production and purification are still required for precise biochemical characterization of top hits. Given the good correlation between datasets, the reaction half‐time proved to be a sufficient measure for our current purposes, and precise determination of catalytic protein activity was not required at this stage.

Ordering of mutants according to their biological activity provides exceptionally valuable information for machine learning–driven protein engineering [25]. Unlike traditional approaches that rely on absolute activity measurements obtained from a limited number of purified variants, ranking‐based datasets capture relative functional trends across large mutant libraries [26, 27]. Importantly, the platform enables systematic data collection across the full functional landscape, including variants with low expression levels or poor stability that cannot be produced in sufficient quantities for conventional biochemical characterization. The inclusion of these low‐performing or inactive mutants generates high‐value “negative” data that are typically missing from enzyme engineering campaigns [26]. Such data are crucial for preventing model bias, improving generalization, and accurately defining functional boundaries within sequence space [25]. The resulting balanced datasets, that is, containing both highly active and poorly performing variants, substantially enhance the model training and validation.

Beyond the specific enzyme system studied here, this approach is broadly applicable to protein engineering, directed evolution, synthetic biology workflows. The ability to rapidly generate balanced functional datasets makes experimental screening platforms powerful data engines for artificial intelligence, accelerating discovery cycles and enabling more reliable exploration of vast protein sequence space. Overall, our approach offers a valuable tool for accelerating protein engineering efforts, particularly when balancing throughput and accuracy.

## Data Accessibility Statement

All data supporting the findings of this study are available from the corresponding author upon request.

## Author contributions

MT performed experiments, analyzed the data, wrote the manuscript, and acquired funding. VH performed experiments and contributed to data analysis and manuscript revision. JD revised the manuscript, and ES revised the manuscript and supervised the study. All authors have read and approved the final version of the manuscript.

## Conflict of interest

The authors declare no conflict of interest.

## Supporting information


**Figure 1** High‐performance liquid chromatography (HPLC) elution profiles of the analyzed SAK variants.
