# Supplementary material for: Rapid screening of staphylokinase protein variants using an unpurified cell‐free expression system
Source: FEBS Open Bio. 2026 Mar 24;16(8):1452–8. doi: 10.1002/2211-5463.70229 (PMC13398934; doi:10.1002/2211-5463.70229)
Supplement: Supplementary file 1 — Figure 1 High‐performance liquid chromatography (HPLC) elution profiles of the analyzed SAK variants. [file FEB4-16-1452-s001.docx]

Supporting information to

**Rapid Screening of Staphylokinase Protein Variants using an Unpurified Cell-free Expression System**

Maria Tomková^1*^, Veronika Hovanová^1^, Jiri Damborsky^3, 4^, Erik Sedlák^1, 2^

*^1^Center for Interdisciplinary Biosciences, P. J. Šafárik University in Košice, Jesenná 5, 04001 Košice, Slovakia*

*^2^Department of Biochemistry, Faculty of Science, P. J. Šafárik University in Košice, Moyzesova 11, 04001 Košice, Slovakia*

*^3^ Loschmidt Laboratories, Department of Experimental Biology and RECETOX, Masaryk University, Brno, Czech Republic*

*^4^ International Clinical Research Centre, St. Anne’s University Hospital, Brno, Czech Republic*

**^*^ To whom correspondence should be addressed:**

Centre for Interdisciplinary Biosciences, P. J. Šafárik University in Košice, Jesenná 5, 04001 Košice, Slovakia; Email: [maria.tomkova@upjs.sk](mailto:maria.tomkova@upjs.sk), Phone: +421 55 234 2242

**
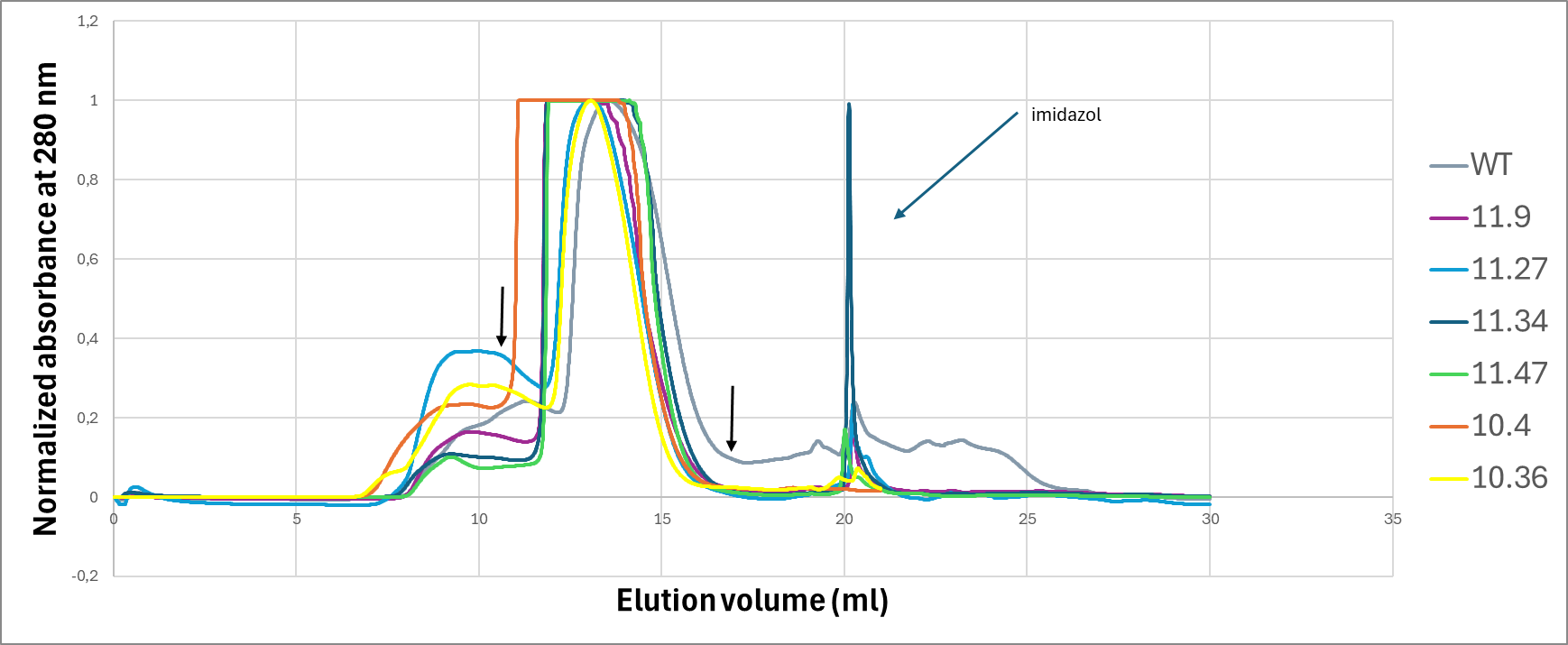
**

**Supplementary Figure 1**. **High-performance liquid chromatography (HPLC) elution profiles of the analysed SAK variants.** Black arrows indicate the collected elution fractions.
